# Перспективы применения свёрточных нейронных сетей в цитологической диагностике узловых образований щитовидной железы

**DOI:** 10.14341/probl13475

**Published:** 2025-07-22

**Authors:** М. В. Солопов, А. С. Кавелина, А. Г. Попандопуло, В. В. Турчин, Р. В. Ищенко, Д. А. Филимонов

**Affiliations:** Институт неотложной и восстановительной хирургии им. В.К. Гусака; Институт неотложной и восстановительной хирургии им. В.К. Гусака; Институт неотложной и восстановительной хирургии им. В.К. Гусака; Институт неотложной и восстановительной хирургии им. В.К. Гусака; Институт неотложной и восстановительной хирургии им. В.К. Гусака; Институт неотложной и восстановительной хирургии им. В.К. Гусака

**Keywords:** щитовидная железа, узловое образование, свёрточная нейронная сеть, искусственный интеллект, тонко­игольная аспирационная биопсия, цитодиагностика

## Abstract

**АКТУАЛЬНОСТЬ:**

АКТУАЛЬНОСТЬ. Цитологическое исследование очаговых образований щитовидной железы (ЩЖ) является золотым стандартом диагностической программы верификации доброкачественных и злокачественных поражений этого органа. Рост заболеваемости, недостаток специалистов и потребность автоматизации медицинской диагностики делают применение машинного обучения, особенно свёрточных нейронных сетей, перспективным направлением в цитологической диагностике патологии ЩЖ.

**ЦЕЛЬ:**

ЦЕЛЬ. Анализ и оценка роли свёрточных нейронных сетей в цитологической диагностике патологии ЩЖ, исследование их потенциала для повышения точности и автоматизации диагностических процессов.

**МЕТОДЫ:**

МЕТОДЫ. Анализ литературы из баз данных Pubmed, Google Scholar и научной электронной библиотеки elibrary.ru с использованием ключевых слов «thyroid», «cytology», «cytopathology», «fine-needle aspiration biopsy», «neural network» и «convolutional neural network». Для анализа отобрано 12 статей, опубликованных с 2018 по 2023 гг.

**РЕЗУЛЬТАТЫ:**

РЕЗУЛЬТАТЫ. В работе рассмотрены основные принципы устройства свёрточных нейронных сетей и показатели, которые используются для оценки их качества. Выполнен анализ исследований по применению свёрточных нейронных сетей в цитологической диагностике патологии ЩЖ. В соответствии с результатами указанные нейронные сети классифицируют патологические состояния с высокой точностью и чувствительностью, сравнимой с работой опытного цитолога. Точность классификации папиллярного рака может достигать 99,7%. Однако отсутствие единых стандартов подготовки изображений для обучения нейронных сетей, недостаточное количество исследований с использованием многоцентровых данных и узкий диагностический спектр имеющихся нейросетевых моделей пока ограничивает внедрение таких систем искусственного интеллекта в цитологическую диагностическую практику.

**ЗАКЛЮЧЕНИЕ:**

ЗАКЛЮЧЕНИЕ. Имеющиеся результаты исследований разнообразных вариантов использования свёрточных нейронных сетей в цитологической диагностике патологии ЩЖ имеют все шансы стать инициатором серьезного сдвига парадигмы привычной цитопатологии в сторону цифровой и вычислительной цитопатологии, в которых основные функции будут выполнять системы на основе искусственного интеллекта.

## Abstract (en)

**AIM:**

AIM. Analysis and assessment of the role of convolutional neural networks in the cytological diagnosis of the thyroid pathology, exploring their potential for increasing the accuracy and automation of diagnostic processes.

**METHODS:**

METHODS. Analysis of literature from Pubmed, Google Scholar and the scientific electronic library elibrary.ru using the keywords «thyroid», «cytology», «cytopathology», «fine-needle aspiration biopsy», «neural network» and «convolutional neural network». 12 articles published from 2018 to 2023 were selected for analysis.

**RESULTS:**

RESULTS. The paper discusses the basic principles of the design of convolutional neural networks and the metrics that are used to assess their quality. An analysis of studies on the use of convolutional neural networks in the cytological diagnosis of the thyroid pathology was performed. According to the results, these neural networks classify pathological conditions with high accuracy and sensitivity, comparable to the work of an experienced cytologist. The accuracy of classification of papillary carcinoma can reach 99.7%. However, the lack of uniform standards for preparing images for training neural networks, the insufficient number of studies using multicenter data, and the narrow diagnostic range of available neural network models still limit the implementation of such AI systems in cytological diagnostic practice.

**CONCLUSION:**

CONCLUSION. The available research results on various options for using convolutional neural networks in the cytological diagnosis of the thyroid pathology have every chance of becoming the initiator of a serious paradigm shift in conventional cytopathology towards digital and computational cytopathology, in which the main functions will be performed by AI systems.

## ВВЕДЕНИЕ

При плановом ультразвуковом исследовании щитовидной железы (ЩЖ) могут выявляться очаговые образования, отличающиеся по сонографическим параметрам от остальной ткани и имеющие четкие границы. Обычно они обнаруживаются у 48% пациентов [1]. При этом по протоколу необходима оценка их размера, расположения, эхогенности, четкости границ и наличия кальцификатов. Однако сами по себе ультразвуковые характеристики несут незначительную информацию о морфологии зоны интереса и, соответственно, степени риска развития неопластического процесса. В связи с этим возникает необходимость морфологической верификации этих образований при помощи цитологического исследования. В большинстве случаев узловые образования ЩЖ являются доброкачественными, однако около 5% имеют онкологическую трансформацию различного характера.

Показаниями к проведению тонкоигольной аспирационной пункционной биопсии (ТАПБ) являются либо сонографические критерии, изложенные в различных модификациях классификации TIRADS [2][3], либо национальные клинические рекомендации по лечению патологии ЩЖ, например, клинические рекомендации «Диффренцированный рак щитовидной железы», утвержденные Минздравом России [4]. Цитологическое исследование ТАПБ щитовидной железы является золотым стандартом обследования, позволяя быстро диагностировать доброкачественные и злокачественные поражения, избавив пациента от ненужного хирургического вмешательства. Точность метода составляет 89–95% [5], чувствительность — 68–98%, специфичность — 56–100% [6]. Однако надежность метода вариабельна, а результат исследования зависит как от квалификации цитолога, так и от качества исследуемого препарата (материал может быть неинформативным из-за затемнения от форменных элементов крови, повреждения или недостаточного количества клеток), что впоследствии затрудняет идентификацию патологий. Для стандартизации в цитологической диагностике опухолевой и неопухолевой патологии ЩЖ используется система классификации Bethesda, которая разделяет результаты исследования на шесть групп различных категорий риска злокачественности образования (I–VI) и определяет дальнейшие действия, например, повторная биопсия, наблюдение или хирургическое вмешательство [7]. В 15–30% результатов диагностики по этой классификации имеется неопределенность в III (атипия фолликулярного эпителия неясного значения) и IV категориях (фолликулярная опухоль, вероятно, аденома или рак), т.е. определении доброкачественности или злокачественности опухоли. Результаты метаанализов свидетельствуют о риске наличия злокачественных новообразований в III и IV категориях в 30,5 и 28,9% соответственно [5][8].

Проведение хирургической резекции образований ЩЖ для окончательного гистологического анализа позволяет с высокой точностью определить характер патологического процесса, но не предполагает возможность принятия предоперационных решений, что подвергает пациента риску развития периоперацинного стресса и последствий самого оперативного вмешательства. Частота хирургической резекции ЩЖ составляет 20–25% для всех типов узловых образований, при этом менее половины из них оказываются действительно злокачественными при окончательном гистологическом исследовании [9]. Чтобы исключить неоправданные хирургические вмешательства, нужны быстрые и точные методы ранней диагностики рака. Стандартная/традиционная цитодиагностика трудозатратна и требует высокой квалификации специалиста-цитолога. Молекулярно-генетические анализы спорных узловых образований ЩЖ внедряются в практическую медицину, но в настоящее время их широкое применение ограниченно из-за недостаточной доступности и дороговизны. Кроме того, они демонстрируют различную чувствительность и специфичность с тенденцией к занижению вероятности наличия онкологической патологии [10]. За последние годы значительно продвинулась разработка и внедрение в цитодиагностику ЩЖ методов искусственного интеллекта (ИИ), которые привнесли новые возможности для повышения эффективности и точности диагностики [11][12].

Основной целью развития систем на основе ИИ является разработка таких компьютерных систем, которые способны решать проблемы, не будучи явно запрограммированными. В связи с накоплением в последние годы наборов данных из различных областей, улучшением вычислительных мощностей и доступностью программных библиотек с открытым исходным кодом, активно развивается и внедряется машинное обучение (МО) — одна из областей ИИ, специалисты которой занимаются разработкой «умных» алгоритмов для выполнения задач регрессии, классификации и кластеризации. Такие алгоритмы по мере обработки больших массивов данных (т.е. процесса обучения) улучшают показатели эффективности решения задачи. На основе маркировки обучающих данных МО подразделяется на контролируемое и неконтролируемое. При контролируемом обучении выходным данным присваивается специальная метка, которая отражает истинную характеристику анализируемого объекта, а входными данными являются параметры этого объекта. После многократного анализа данных с метками модель может правильно характеризовать новые входные данные без меток. В неконтролируемом обучении не требуется использование меток, т.е. алгоритм сам старается найти зависимости в представленных данных. Примером неконтролируемого обучения является кластеризация подгрупп рака молочной железы на основе гистологических признаков [13].

Одной из самых популярных областей МО является применение искусственных нейронных сетей (ИНС) в рамках так называемого глубокого обучения. Такие системы называют «глубокими», потому что они состоят из множества слоев, в каждый из которых включено определенное количество вычислительных единиц (нейронов). ИНС успешно решают задачи по анализу изображений и видео, распознаванию речи. В биомедицине ИНС находят применение для исследования взаимодействия разрабатываемых лекарств с потенциальными мишенями, прогнозирования побочных эффектов от лекарств, автоматизации интерпретации медицинских данных, прогнозирования госпитализации и смертности [14]. В диагностике заболеваний ЩЖ развиваются методы МО на основе анализа данных ультразвуковых, радиологических и цитологических исследований [15].

В этой работе будет рассмотрено использование сверточных нейронных сетей (СНС) для цитологической диагностики патологии ЩЖ. Такие нейронные сети хорошо распознают формы и текстуры на изображениях и выполняют классификацию с высокой точностью и чувствительностью. СНС автоматически анализируют важные детали изображений (признаки) без необходимости указания со стороны человека, на что обращать внимание, что делает процесс идентификации и классификации изображений более эффективным. Использование СНС в цитологической диагностике в будущем может снизить рабочую нагрузку цитологов, ускоряет сортировку и анализ изображений, выделяя наиболее значимые для специалиста случаи. Далее будут рассмотрены основы строения СНС, принцип их функционирования и наиболее распространенные показатели, используемые при классификации изображений.

## МАТЕРИАЛЫ И МЕТОДЫ

С использованием комбинирования группы ключевых слов «thyroid», «cytology», «cytopathology», «fine-needle aspiration biopsy» с группой «neural network», «convolutional neural network» и их аналогов на русском языке нами выбрана и проанализирована литература из таких баз данных: Pubmed, Google Scholar и научная электронная библиотека elibrary.ru. Изначальная выборка содержала 42 статьи, из которых затем отбирались только те исследования, в которых применялись СНС для анализа цитологических изображений ЩЖ. Для анализа отобрано 12 статей, все из которых были опубликованы в период с 2018 по 2023 гг. Изучены аннотации и полнотекстовые версии публикаций.

## ОСНОВЫ СНС

Основа для развития СНС была заложена экспериментальными исследованиями структуры зрительной коры головного мозга кошек и обезьян, которые проводили Дэвид Х. Хьюбел и Торстен Визель в 1950–60-х гг. За эти исследования они были удостоены Нобелевской премии по физиологии и медицине в 1981 г. [16]. Исследования показали, что многие нейроны зрительной коры обладают небольшими локальными рецептивными полями и активируются только при наличии стимулов в определенной области поля зрения. Перекрытие рецептивных полей различных нейронов обеспечивает покрытие всего поля зрения. Хьюбел и Визель обнаружили, что нейроны могут иметь одно и то же рецептивное поле, но отвечать на различные простые геометрические формы. Более того, были выявлены нейроны с более сложными рецептивными полями, реагирующие на комбинации базовых образов. Эти наблюдения привели к созданию модели организации зрительных нейронов, в которой нейроны более высокого уровня используют выходы соседних нейронов более низкого уровня.

Остановимся на том, как работают СНС (рис. 1), анализируя изображения на наличие определенных признаков. Это достигается путем применения набора фильтров к изображению, каждый из которых «смотрит» на определенный тип особенностей, таких как края, углы, текстуры или более сложные паттерны. Фильтр — это математическая матрица небольшого размера (например, 3x3 или 5x5), которая содержит весовые коэффициенты, определяющие, какие признаки фильтр будет извлекать из изображения. По сути, фильтр является моделью рецептивного поля нейрона. Фильтр перемещается по растровой сетке изображения и на каждой позиции определяет характерные паттерны из соответствующего участка изображения. Этот процесс называется свёрткой. После сканирования изображения каждым фильтром создается карта признаков. После свёртки следует процесс пулинга (pooling), который обеспечивает уменьшение пространственного объема карты признаков с селекцией наиболее значимых. Также пулинг помогает повысить устойчивость нейронной сети к незначительным изменениям в изображениях, таким как смещения или искажения. Операции свёртки и пулинга повторяются многократно, каждый раз все более уточняя и конкретизируя признаки, которые сеть может распознать. Карта признаков, полученная после операций свёртки и пулинга, представляет собой многомерный массив (тензор) числовых значений пикселей, который на финальном этапе извлечения признаков преобразуется в одномерный массив путем процедуры разворачивания многомерного массива (flattening). Затем этот массив передается в нейронную сеть с полносвязными слоями, которая выполняет классификацию изображения. В полносвязном слое каждый нейрон (его математическая модель) соединен со всеми нейронами предыдущего слоя. Значимость каждой связи определяется весовым коэффициентом. На каждом полносвязном слое массив признаков умножается на матрицу весовых коэффициентов, а результат передается функции активации, которая формирует нелинейные связи в нейронной сети, что способствует обнаружению сложных зависимостей данных. Последний полносвязный слой, называемый выходным слоем, создает конечные выходные значения сети, представляющие собой вероятности классов изображений (рак или доброкачественное узловое образование).

**Figure:**
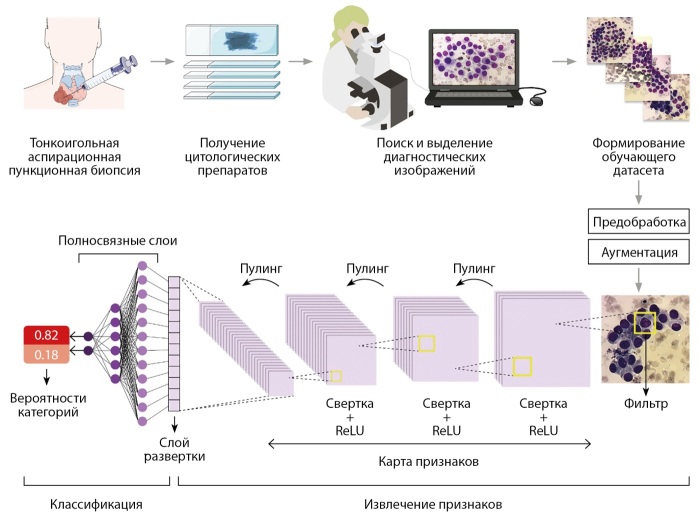
Рисунок 1. Алгоритм обучения СНС для цитологической диагностики патологии щитовидной железы.

Обучение СНС происходит с использованием метода, известного как обратное распространение ошибки. Сначала через сеть проходят данные изображения и делается прогноз. Затем выполняется сравнение прогноза сети и истинного значения, и на основе этого сравнения вычисляется ошибка. После этого ошибка распространяется обратно по сети, корректируя весовые коэффициенты связей между нейронами таким образом, чтобы минимизировать ошибку. Этот процесс повторяется множество раз на большом наборе обучающих данных, позволяя сети «научиться» распознавать и классифицировать изображения. Несмотря на то, что нейронные сети демонстрируют точные результаты, при обсуждении проблем их использования встречается понятие «черный ящик» [17]. Оно характеризует непонятный для человека процесс извлечения признаков внутри слоев нейронной сети путем сложных нелинейных операций над входными данными и отсутствие обоснования получаемых прогнозов.

Ключевым моментом для успешного обучения СНС является объем выборки тренировочных изображений. В зависимости от конкретной задачи, для решения которой будет использоваться обученная СНС, этот объем будет отличаться в зависимости от доступности данных и сложности паттернов классифицируемых изображений. По мнению Bakaev и соавт. [18], для эффективного обучения СНС должен использоваться датасет размером не менее 17 тыс. изображений. В случае применения СНС для классификации микрофотографий цитологических препаратов ЩЖ использовались датасеты размером от нескольких сотен [19] до 150 тыс. [11] изображений.

При использовании СНС в цитологической диагностике патологии ЩЖ распространенной практикой является извлечение изображений диагностических областей из цельнопрепаратных изображений (ЦПИ), полученных с помощью специального сканирующего оборудования [11]. Такой подход позволяет получить достаточного много изображений, подходящих для обучения СНС. Для расширения размера выборки изображений часто применяется аугментация — процесс искусственного увеличения разнообразия изображений посредством применения к ним различных трансформаций: поворотов, отражений, изменения масштаба, сдвигов, изменений яркости и контраста и т.д.

Для характеристики успешности обучения СНС используют ряд показателей, которые оценивают производительность модели. В рамках данной работы мы приводим характеристики основных показателей, которые используются в рассматриваемых далее исследованиях по применению СНС в цитологической диагностике патологии ЩЖ (табл. 1).

**Table:** Таблица 1. Основные показатели, используемые для оценки производительности СНС Примечание. TP — количество истинных положительных прогнозов, TN — количество истинных отрицательных прогнозов, FP — количество ложных положительных прогнозов, FN — количество ложных отрицательных прогнозов, p — точность (precision), r — полнота (recall). В машинном обучении за положительный случай принимается наиболее редкое явление, а более частое — за отрицательный.

Показатель	Формула вычисления	Описание
Общая точность (accuracy)		Доля правильных прогнозов по отношению ко всем выполненным прогнозам
Точность (precision)		Доля правильных положительных прогнозов по отношению ко всем положительным прогнозам
Полнота (recall) / чувствительность (sensitivity)		Доля правильных положительных прогнозов по отношению ко всем положительным случаям
Специфичность (specificity)		Доля правильных отрицательных прогнозов по отношению ко всем истинным отрицательным случаям
Оценка F1(F1-score)		Среднее гармоническое значение точности и чувствительности
AUC(area under curve)	–	Площадь под графиком, который иллюстрирует соотношение между чувствительностью и специфичностью модели при разных порогах классификации. Значение варьирует от 0,5 (случайное угадывание) до 1 (идеальная модель)

Для конкретной задачи специалистами в области МО могут создаваться собственные уникальные архитектуры СНС, однако имеется уже ряд разработанных моделей, которые хорошо зарекомендовали себя для классификации цитологических изображений: EfficientNet, Inception, VGG, U-Net, SegNet, DenseNet, ResNet, GoogleNet, AlexNet [20]. Эти архитектуры предварительно обучены на естественных изображениях из базы данных ImageNet. Они точно и быстро обнаруживают признаки клеточной атипии на цитологических снимках, например, сеть AlexNet показала свою эффективность в выявлении рака шейки матки с точностью 99% [21].

## ПРИМЕНЕНИЕ СНС В ЦИТОЛОГИЧЕСКОЙ ДИАГНОСТИКЕ ПАТОЛОГИИ ЩИТОВИДНОЙ ЖЕЛЕЗЫ

В последние годы был выполнен ряд исследований по применению СНС для классификации патологии ЩЖ, продемонстрировавших обнадеживающие результаты, которые могут заложить основу для разработки интеллектуальных инструментов для раннего выявления злокачественных новообразований. В таблице 2 представлена сводная информация по последним исследованиям в этом направлении.

**Table:** Таблица 2. Исследования по использованию СНС для цитологической диагностики щитовидной железы Примечание. Acc. — общая точность (accuracy), Sen. — чувствительность (sensitivity), Spec. — специфичность (specificity), Prec. — точность (precision), Rec. — полнота (recall), F1 — оценка F1, AUC — площадь под ROC-кривой, MTC — медуллярный рак щитовидной железы, PDTC — низкодифференцированный папиллярный рак щитовидной железы, ТАПБ — тонкоигольная аспирационная пункционная биопсия.

Год / ссылка	Цель	Тип СНС	Датасет	Результаты
2018 / Sanyal и соавт. [19]	Бинарная классификация папиллярного рака	Модель разработана авторами	370 изображений из 20 препаратов (184 — папиллярный рак, 186 — другие патологии)	Aсс. — 85,1% Sen. — 90,5% Spec. — 83,3%
2019 / Guan и соавт. [22]	Бинарная классификация папиллярного рака	VGG-16, Inception-v3	887 изображений из 279 препаратов (476 — папиллярный рак, 411 — доброкачественные)	Acc. — 97,7% Sen. — 100% Spec. — 94,9%
2020 / Elliott Range и соавт. [25]	Классификация злокачественных и доброкачественных поражений	2 СНС на основе VGG11	1000 изображений из 908 препаратов	Sen. — 92% Spec. — 90,5% AUC — 0,932
2022 / Dov и соавт. [26]	Оценка согласованности ИИ-системы с работой цитолога	2 СНС на основе VGG11	100 изображений из последовательных 109 ТАПБ	Коэффициент статистики Коэна (κ) 0,924
2022 / Duan и соавт. [27]	Классификация злокачественных и доброкачественных поражений	YOLOv4, EfficientNet	29 780 изображений для модели обнаружения и 63 356 изображений для модели классификации из 360 препаратов	Acc. — 97,9% Prec. — 97,8% Rec. — 98,1% F1 — 97,9%
2022 / Duc и соавт. [28]	Бинарная классификация папиллярного рака	DenseNet	367 изображений (222 — папиллярный рак, 145 — доброкачественные)	Acc. — 99,7% Sen. — 97,3% Spec. — 94,1%
2023 / Alabrak и соавт. [17]	Классификация фолликулярной аденомы и фолликулярного рака	Модель разработана авторами	886 изображений из 43 клинических случаев (527 изображений фолликулярной аденомы и 359 изображений фолликулярного рака)	Acc. — 78% Sen. — 88,4% Spec. — 64% AUC — 0,87
2023 / Hirokawa и соавт. [11]	Классификация 9 типов патологий щитовидной железы	EfficientNet	148 395 изображений из 393 узловых образований щитовидной железы	Для большинства категорий AUC>0,95 (кроме MTC и PDTC)
2023 / Jang и соавт. [31]	Распознавание неинформативных препаратов ТАПБ	FNA-Net, разработана авторами	287 изображений от 6 пациентов	F1 — 0,81 AUC — 0,84

Sanyal и соавт. разработали СНС для классификации папиллярного рака ЩЖ по микрофотографиям препаратов ТАПБ [19]. Из 20 цитологических препаратов (20 пациентов), полученных от двух разных медицинских центров Северной Индии, был сформирован датасет из 370 изображений с разрешением 512x512 пикселей, каждое из которых представляло область диагностического интереса. Для обучения СНС датасет был разделен на два класса: 184 цитологических изображения папиллярного рака, 186 изображений доброкачественных новообразований (коллоидного зоба, фолликулярных новообразований и лимфоцитарного тиреоидита). В исследование включали только цитологически подтвержденные случаи папиллярного рака, пограничные случаи не использовались. Для исследования обобщающей способности СНС анализировать данные, полученные разнородным образом, микрофотографии для формирования датасета были сделаны на двух моделях микроскопов с использованием объективов с разными увеличениями (10x и 40x). Наилучших показателей сеть достигла, когда общий прогноз о наличии рака формировался после комбинированного анализа фотографий, сделанных при двух увеличениях: общая точность — 85,1%, чувствительность — 90,5% и специфичность — 83,3%. Тем не менее авторы указывают на недостатки сети, когда расплывчатые папиллярные образования фолликулярных клеток ошибочно идентифицируются как папиллярный рак.

Guan и соавт. [22] использовали две архитектуры СНС VGG-16 и Inception-v3 для классификации папиллярного рака. Для исследования использовали 279 цитологических препаратов, полученных методом жидкостной цитологии, из которых 159 характеризовались как папиллярный рак, а 120 были охарактеризованы как неонкологическая патология. Все случаи рака по системе классификации Bethesda имели классы V или VI c гистологическим подтверждением. Доброкачественные случаи имели класс II, но так как пациентам не проводилась хирургическая операция, гистологического подтверждения не было. Исходные изображения были получены путем оцифровки препаратов при 400x увеличении. Затем изображения нарезали на фрагменты размером 224x224 пикселя, которые содержали фолликулярные клетки. В итоге датасет содержал 887 изображений (476 случаев рака и 411 доброкачественных новообразований). Посредством отражений и вращений изображений датасет был увеличен в восемь раз и разделен на обучающую и тестовую группы в соотношении 6:1. Из двух рассматриваемых архитектур СНС наилучшие показатели продемонстрировала VGG-16: общая точность — 97,7% (95% на уровне пациентов), чувствительность — 100% и специфичность — 94,9%. Дополнительно авторы исследовали характеристики клеточных ядер, которые автоматически извлекали из изображений и сравнивали по категориям. Полученные численные показатели, отражающие количество контуров, периметр и площадь клеток, а также средняя интенсивность пикселей были статистически больше у злокачественных клеток.

Заслуживает внимания цикл работ группы исследователей из США по автоматизации диагностики патологии ЩЖ. Dov и соавт. высказали идею о том, что Для цитологической диагностики узловых образований ЩЖ может использоваться тандем нейронных сетей [23]. Сначала идентифицируются области на ЦПИ, содержащих группы фолликулярных клеток, а затем эти области используются для прогнозирования злокачественных новообразований по категориям Bethesda [24]. Реализация этого подхода была изложена в работе Elliott Range и соавт. [25], в которой исследователи разработали систему из двух СНС на основе архитектуры VGG11. Первая сеть использовалась для определения на изображениях информативных областей фолликулярных клеток; вторая сеть анализировала эти области и прогнозировала классификационную категорию и ассоциированный прогноз о доброкачественности или злокачественности новообразований. Обучающие изображения были сформированы из 908 биопсий (от 659 пациентов) с гистологическим подтверждением, окрашенных по Папаниколау. Для обучения первой СНС патологоанатом вручную выделял области диагностического интереса, неинформативные участки выделялись случайным образом, так как большая часть площади ЦПИ не содержит клеточного материала. С использованием первой СНС из изображений было извлечено 1000 областей с наивысшей степенью информативности, которые впоследствии использовалась для обучения второй СНС, выполняющей роль классификатора. Система достигла общей точности 90,8%, чувствительности 92,0%, специфичности 90,5% и AUC 0,932. Последний показатель был сопоставим с уровнем первоначального диагноза цитолога (0,931). Для улучшения точности прогнозов злокачественности новообразований было создано правило, комбинирующее решения алгоритма СНС и цитолога. Алгоритм предоставлял прогнозы только для случаев, изначально классифицированных специалистом как неопределенные поражения, которые не являются доброкачественными или злокачественными и относятся к категориям III и IV по классификации Bethesda [7], а именно: атипия фолликулярного эпителия неясного значения, фолликулярная опухоль и подозрение на фолликулярную опухоль. Это привело к увеличению AUC с 0,931 до 0,962 и повышению специфичности с 90,5 до 92,9%. По мнению авторов, использование систем СНС в качестве вспомогательного инструмента для цитологической диагностики способствует устранению неоднозначностей при рассмотрении трудно классифицируемых случаев патологии ЩЖ [12].

На основе полученных результатов этот же коллектив [26] разработал программу для выделения информативных участков и диагностики по ЦПИ и протестировал ее в сравнении с работой цитолога. Для этого цитолог просматривал ЦПИ от 109 последовательных ТАПБ ЩЖ и записывал диагноз. В то же время алгоритм программы проводил скрининг и отбирал 100 информативных участков из каждого изображения, которые спустя 117 дней демонстрировались специалисту для постановки диагноза. Оценка соответствия между диагнозами с помощью метода κ-статистики Коэна продемонстрировала высокий уровень совпадений между диагнозами (κ=0,924). Среднее время, затраченное на постановку диагноза с использованием программы, составило 81,6 секунды, что указывает на потенциальную перспективность использования этого инструмента для снижения рабочей нагрузки цитолога. Однако недостатком этого исследования является участие в нем только одного цитолога.

Duan и соавт. предложили систему автоматизированной диагностики на основе двух модулей СНС для автоматического скрининга рака щитовидной железы по ЦПИ [27]. Для обучения нейронных сетей исследователи собрали 360 препаратов жидкостной цитологии ТАПБ из нескольких медицинских учреждений. Из исходного набора препаратов формировали два датасета изображений: первый — для обнаружения диагностических областей, второй — для бинарной классификации по категориям системы Bethesda (II — доброкачественные, III–VI — злокачественные). Для обнаружения диагностических областей использовалась архитектура YOLOv4 — производная сеть четвертого поколения сети YOLO, нацеленная на оптимальный баланс между количеством свёрточных слоев и количеством параметров. В качестве классификатора использовалась архитектура EfficientNet, которая обеспечивает более высокие значения точности по сравнению с другими моделями, благодаря использованию масштабирования по глубине, ширине и разрешению. После классификации система демонстрировала врачу 10 изображений с наибольшей вероятностью злокачественности. На обучающем наборе классификационный модуль достиг общей точности 97,9%, точности 97,8%, оценки F1 97,9%, полноты 98,1%. При этом на тестовом наборе изображений модуль EfficientNet продемонстрировал точность 78,7%, а в комбинации с YOLOv4 — 81,8%. Этот результат показывает способность предлагаемой системы избегать игнорирования истинно отрицательных прогнозов, когда случаи похожи на положительные.

Перспективная работа была проведена Alabrak и соавт., которые использовали собственно разработанную архитектуру СНС для диагностирования патологий ЩЖ IV категории Bethesda [17]. Эта категория включает как фолликулярную аденому, так и фолликулярный рак. Эти патологии сложно отличить друг от друга цитологическими методами, поэтому требуется резекция для проведения гистологического исследования и определения капсулярного и лимфо-васкулярного состояния опухоли. Так как фолликулярные аденомы клинически безвредны, особенную диагностическую ценность представляет выявление фолликулярного рака. Для обучения модели использовали 886 изображений, из которых 527 изображений (28 клинических случаев) характеризовали фолликулярную аденому, а 359 изображений (15 клинических случаев) — фолликулярный рак. Обученная модель при дифференциации фолликулярной аденомы от фолликулярного рака достигла общей точности 78,0%, чувствительности 88,4%, специфичности 64% и AUC 0,87.

Hirokawa и соавт. на момент написания статьи провели самое масштабное исследование по применению СНС для диагностики 9 типов патологии ЩЖ: доброкачественных образований, анапластического рака, фолликулярной аденомы, фолликулярного рака, медуллярного рака, оксифильной фолликулярной опухоли, папиллярного рака, низкодифференцированного рака и лимфомы ЩЖ [11]. Для обучения СНС на основе архитектуры EfficientNetV2-L использовали 148 395 фрагментированных изображений мазков ТАПБ из 393 узловых образований. Большинство категорий модель эффективно различала (AUC>0,95), однако при оценке низкодифференцированного и медуллярного рака показатели были ниже: 0,49 и 0,91 соответственно. Системе было трудно отличить низкодифференцированный онкологический процесс от папиллярного, медуллярного и фолликулярного рака, показатель полноты для этого случая был самым низким (35,4%). Также была выполнена правильная классификация для 16 из 35 новообразований, которые изначально были отнесены к группе атипии неопределенной значимости. При классификации злокачественности узловых образований этой группы показатели чувствительности, специфичности, положительной прогностической ценности и отрицательной прогностической ценности составили 94,4%, 15,4%, 60,7% и 66,7% соответственно. Отдельного внимания заслуживает возможность разработанной модели отличать фолликулярную аденому от фолликулярного рака, которые обычно дифференцируют по гистологическим и клиническим признакам. Показатель полноты для указанных патологий составил 86,7 и 93,9% соответственно. Несмотря на ограничения и недостатки разработанной системы, включающей снижение специфичности для узловых образований с неопределенными атипиями и худшие показатели для низкодифференцированных форм рака, полученные авторами результаты демонстрируют возможности применения систем на основе СНС для классификации широкого спектра патологий ЩЖ.

Наилучшие показатели точности прогнозирования папиллярного рака с применением СНС продемонстрировали в своем исследовании Duc и соавт. [28]. Цитологические изображения препаратов жидкостной цитологии ThinPrep автоматическим методом были разбиты на фрагменты, аннотированы двумя опытными цитологами, а затем разделены на обучающие, валидационные и тестовые подгруппы. Для формирования датасета использовали 367 оригинальных изображений, из которых 222 случая папиллярного рака и 145 случаев доброкачественных новообразований. Для классификации изображений сравнивали эффективность нескольких типов архитектур СНС: ResNet, DenseNet и Inception. Наилучшие показатели классификации рака по цифровым изображениям тестового набора были достигнуты с использованием архитектуры DenseNet161: общая точность — 95,6%, чувствительность — 97,3%, специфичность — 94,1%. За счет применения метода ансамблевого обучения путем комбинации с несколькими моделями СНС классификатора AdaBoost удалось повысить точность классификации до 99,7%. Повышение точности прогнозирования также было достигнуто посредством цветовой нормализации изображений по методу Рейнхарда [29].

Модели на основе СНС также могут использоваться для распознавания неинформативных препаратов ТАПБ, частота встречаемости которых может варьировать от 3 до 38% [30]. Jang и соавт. первыми разработали ансамблевую модель FNA-Net, которая позволяет in situ проводить оценку неокрашенных мазков аспиратов ткани ЩЖ для выявления препаратов, которые не несут диагностической ценности [31]. Система состоит из двух моделей глубокого обучения для обнаружения кластеров фолликулярных клеток: классификатора ЦПИ и сети Faster R-CNN. Датасет состоял из 205 обучающих изображений, по 41 изображению в валидационной и тестовой выборках. Для проверки способности модели идентифицировать полезные для диагностики препараты авторы создали 10 000 дополнительных наборов данных из исходной тестовой выборки путем многократного случайного отбора групп (по 41 изображению от 6 пациентов). Авторам удалось достичь оценки F1 0,81 и AUC 0,84 при обнаружении недиагностических препаратов, на которых содержалось меньше шести клеточных кластеров. Более высокие показатели достигались, когда порог достоверности был повышен до десяти кластеров — оценка F1 0,915 и AUC 0,953.

## ОБСУЖДЕНИЕ

По прогнозам, к 2030 г. рак щитовидной железы может стать четвертым по распространенности среди онкологических заболеваний [32]. Разработка новых эффективных методов ранней диагностики папиллярного рака ЩЖ, как одной из ведущих причин оперативных вмешательств при онкологической патологии во многих странах, является актуальным и перспективным направлением для обеспечения оптимального лечения пациентов. В последние годы разработка и применение методов ИИ в медицинской диагностике значительно продвинулись, создавая новую парадигму цифровой и вычислительной цитопатологии [15]. Традиционная практика использования светового микроскопа и ручного анализа препаратов, остававшаяся неизменной на протяжении последнего века, начала трансформироваться под влиянием этих инноваций. В данном исследовании мы проанализировали случаи использования СНС для классификации цитологических изображений ЩЖ. Во всех исследованиях СНС классифицируют патологические состояния ЩЖ с высокой точностью и чувствительностью, сравнимой с работой опытного цитолога. Тем не менее имеется ряд проблем, изложенных ниже, которые ограничивают внедрение СНС в клиническую практику.

В первую очередь, необходима стандартизация методик получения датасетов обучающих изображений для корректного сравнения эффективностей разных нейросетевых моделей. В некоторых работах получение диагностических фрагментов осуществлялось ручным способом [19][22], однако для получения больших датасетов этот метод является трудоемким. Более перспективным способом является разработка интеллектуальных систем, в которых получение диагностических изображений будет происходить автоматическим способом (за счет возможности применения СНС для сегментации изображений) с последующей их диагностикой в нейросетевом модуле, ответственном за классификацию [26]. Для реализации такого подхода необходимо внедрение в работу организаций здравоохранения оборудования для сканирования цитологических препаратов и разработки программного обеспечения, которое из ЦПИ сможет выделять релевантные изображения для выполнения непосредственно диагностического этапа анализа.

Еще одним существенным недостатком текущих результатов использования СНС для цитологической диагностики является малое количество исследований, в которых обучающие изображения формировались из разных учреждений. Это может быть причиной неточных результатов при трансляции модели в других организациях, поскольку методики подготовки препаратов и изображений могут отличаться. Для решения этой проблемы необходимо проводить исследования с использованием многоцентровых наборов данных, которые будут включать изображения из различных лечебных учреждений и разнообразных групп пациентов. Для успешного развития данного направления необходимо, чтобы каждое учреждение, в котором выполняется цитологическая диагностика, имело базу данных цифровых фотографий цитологических препаратов.

Большинство исследований СНС в цитологической диагностике патологии ЩЖ было связано с классификацией папиллярного рака, фокусируясь на разделении препаратов на две категории: доброкачественные и злокачественные. Однако подобный фокус на бинарной классификации ограничивает способность моделей СНС различать другие, менее распространенные, но серьезные патологии ЩЖ: фолликулярные неоплазии, медуллярные и анапластические формы рака и другие атипичные или доброкачественные образования. Дальнейшие исследования должны стремиться к созданию более сложных моделей, способных классифицировать разные состояния ЩЖ, что позволит клиницистам принимать более обоснованные решения о лечении и последующем наблюдении пациентов.

Перед внедрением технологий на основе СНС в практику цитологической диагностики необходимо провести тщательную оценку их эффективности, переносимости и свободы от предвзятости. Процесс внедрения должен быть постепенным и поэтапным, даже после подтверждения эффективности СНС на тестовых наборах данных, чтобы обеспечить надлежащее доверие к этой технологии со стороны клиницистов и регулирующих органов. Для успешного и безопасного использования таких систем в медицинской практике будущие исследования должны быть направлены на изучение того, как они будут влиять на принятие решений врачами, учитывая как возможные улучшения точности диагностики, так и потенциальные риски. Эти исследования помогут создать безопасные и эффективные интеллектуальные решения, которые ускорят процессы диагностики патологии ЩЖ.

## ЗАКЛЮЧЕНИЕ

В представленной работе мы рассмотрели значимые достижения последних лет в области применения СНС в цитологической диагностике патологии ЩЖ. Несмотря на то, что исследований в этом направлении пока немного, но все они демонстрируют высокую эффективность применения СНС для сегментации и классификации цитологических изображений. Полученные результаты имеют все шансы послужить инициатором серьезного сдвига парадигмы привычной цитопатологии в сторону цифровой и вычислительной цитопатологии. Для более быстрого внедрения интеллектуальных инструментов на основе СНС в клиническую практику необходимо проведение дополнительных многоцентровых исследований, которые помогут разрешить проблемы стандартизации изображений и расширения диагностического спектра нейросетевых моделей.

## ДОПОЛНИТЕЛЬНАЯ ИНФОРМАЦИЯ

Источники финансирования. Работа выполнена по инициативе авторов без привлечения финансирования.

Конфликт интересов. Авторы декларируют отсутствие явных и потенциальных конфликтов интересов, связанных с содержанием настоящей статьи.

Участие авторов. Солопов М.В. — разработка концепции исследования, анализ литературы, написание основного текста статьи, подготовка рисунков; Кавелина А.С. — разработка концепции исследования, анализ литературы; Попандопуло А.Г. — написание вводной части статьи, научное консультирование; Турчин В.В., Ищенко Р.В., Филимонов Д.А. — научное консультирование и редактирование статьи. Все авторы одобрили финальную версию статьи перед публикацией, выразили согласие нести ответственность за все аспекты работы, подразумевающую надлежащее изучение и решение вопросов, связанных с точностью или добросовестностью любой части работы.
